# Artificial Intelligence Compared to Radiologists for the Initial Diagnosis of Prostate Cancer on Magnetic Resonance Imaging: A Systematic Review and Recommendations for Future Studies

**DOI:** 10.3390/cancers13133318

**Published:** 2021-07-01

**Authors:** Tom Syer, Pritesh Mehta, Michela Antonelli, Sue Mallett, David Atkinson, Sébastien Ourselin, Shonit Punwani

**Affiliations:** 1 Centre for Medical Imaging, Division of Medicine, Bloomsbury Campus, University College London, London WC1E 6DH, UK; t.syer@ucl.ac.uk (T.S.); sue.mallett@ucl.ac.uk (S.M.); d.atkinson@ucl.ac.uk (D.A.); 2 Department of Medical Physics and Biomedical Engineering, Faculty of Engineering Sciences, Bloomsbury Campus, University College London, London WC1E 6DH, UK; pritesh.mehta.17@ucl.ac.uk; 3 School of Biomedical Engineering & Imaging Sciences, Faculty of Life Sciences and Medicine, St Thomas’ Campus, King’s College London, London SE1 7EH, UK; michela.antonelli@kcl.ac.uk (M.A.); sebastien.ourselin@kcl.ac.uk (S.O.)

**Keywords:** artificial intelligence, computer-aided diagnosis, machine learning, deep learning, magnetic resonance imaging, PRISMA-DTA, prostate cancer, QUADAS-2, systematic review

## Abstract

**Simple Summary:**

Radiologists interpret prostate multiparametric magnetic resonance imaging (mpMRI) to identify abnormalities that may correspond to prostate cancer, whose status is later confirmed by MR-guided targeted biopsy. Artificial intelligence algorithms may improve the diagnostic accuracy achievable by radiologists alone, as well as alleviate pressures on the prostate cancer diagnostic pathway caused by rising case incidence and a shortage of specialist radiologists to read prostate mpMRI. In this review article, we considered studies that compared the diagnostic accuracy of radiologists, artificial intelligence algorithms, and where possible, a combination of the two. Our review found insufficient evidence to suggest the clinical deployment of artificial intelligence algorithms at present, due to flaws in study designs and biases caused by performance comparisons using small, predominantly single-center patient cohorts. Several recommendations are made to ensure future studies bear greater clinical impact.

**Abstract:**

Computer-aided diagnosis (CAD) of prostate cancer on multiparametric magnetic resonance imaging (mpMRI), using artificial intelligence (AI), may reduce missed cancers and unnecessary biopsies, increase inter-observer agreement between radiologists, and alleviate pressures caused by rising case incidence and a shortage of specialist radiologists to read prostate mpMRI. However, well-designed evaluation studies are required to prove efficacy above current clinical practice. A systematic search of the MEDLINE, EMBASE, and arXiv electronic databases was conducted for studies that compared CAD for prostate cancer detection or classification on MRI against radiologist interpretation and a histopathological reference standard, in treatment-naïve men with a clinical suspicion of prostate cancer. Twenty-seven studies were included in the final analysis. Due to substantial heterogeneities in the included studies, a narrative synthesis is presented. Several studies reported superior diagnostic accuracy for CAD over radiologist interpretation on small, internal patient datasets, though this was not observed in the few studies that performed evaluation using external patient data. Our review found insufficient evidence to suggest the clinical deployment of artificial intelligence algorithms at present. Further work is needed to develop and enforce methodological standards, promote access to large diverse datasets, and conduct prospective evaluations before clinical adoption can be considered.

## 1. Introduction

International guidelines recommend multiparametric magnetic resonance imaging (mpMRI) for biopsy naïve men with suspected prostate cancer for lesion localization prior to MR-guided targeted biopsies [1,2]. Predominantly, radiologists interpret and report mpMRI using the Prostate Imaging-Reporting and Data System (PI-RADS) [3] or Likert-impression scale [4]; sensitivities ranging between 81–90% and specificities ranging between 64–81% have previously been reported for clinically significant prostate cancer detection on mpMRI, by radiologists, in treatment-naïve men [5]. Crucially, mpMRI followed by MR-guided targeted biopsy improves the detection of clinically significant prostate cancer and reduces the over-diagnosis of clinically insignificant prostate cancer, compared to non-targeted transrectal ultrasound-guided (TRUS) biopsies [6]. However, improvements to the prostate cancer diagnostic pathway are needed to identify the small proportion of men whose clinically significant prostate cancer is missed by radiologists reading mpMRI, to reduce the large number of men who undergo unnecessary biopsies due to false positives on mpMRI, and to increase the inter-observer agreement between radiologists of varying experience [5,7,8,9].

Computer-aided diagnosis (CAD) systems that use artificial intelligence (AI) are actively being researched for use in a variety of medical image analysis tasks [10]. The most common roles performed by CAD systems for MRI-based prostate cancer diagnosis are in lesion classification, lesion detection and segmentation, and patient classification [11]. Provided clinical efficacy of systems can be demonstrated, clinical deployment to the prostate cancer diagnostic pathway can be envisioned as (i) companion systems for radiologists during their clinical read, (ii) second reader systems that provide an independent diagnosis, or (iii) patient triage systems that create a clinical workflow based on patient risk. In addition to anticipated improvements in diagnostic accuracy and reporting consistency between readers/centers, CAD systems can alleviate pressures caused by rising case incidence and a shortage of specialist radiologists to read prostate mpMRI [12].

Earlier reviews of CAD systems for MRI-based prostate cancer diagnosis have focused on the technical aspects and potential applications of systems [11,13]. By contrast, this systematic review considers whether sufficient evidence exists to suggest clinical deployment of CAD for prostate MRI. In order to translate systems from research to clinical use, they must demonstrate an advantage over current clinical practice and provide enhanced clinical outcomes. Therefore, clinical readiness of CAD systems should be determined through comparison of their performances to the performance of radiologists, who are the current clinical standard. Accordingly, the key selection criteria for study inclusion in this systematic review is reported radiologist performance to which the performance of CAD is compared.

Our review found insufficient evidence to suggest the clinical deployment of AI CAD systems for prostate MRI, at present, due to methodological flaws in studies identified using quality assessment frameworks, and biases caused by performance comparisons using small, predominantly single-center patient cohorts. While several studies reported superior performance for CAD over radiologist interpretation on small, internal patient datasets, this was not observed in the few studies that performed evaluation using external patient data. Our review concludes that further work is needed to develop and enforce methodological standards, promote access to large diverse datasets, and conduct prospective evaluations before clinical adoption can be considered.

## 2. Materials and Methods

This review was carried out according to the preferred reporting items for systematic review and meta-analysis of diagnostic test accuracy studies (PRISMA-DTA) guidance [14] and performed by both clinical experts and algorithm developers to ensure accurate analysis and sufficient critique of the information presented in studies.

### 2.1. Literature Search

A systematic search of the literature was undertaken by two reviewers independently that included both a clinician and an algorithm developer with an interest in prostate MRI CAD. The search was performed within the MEDLINE, EMBASE, and arXiv electronic databases, and the OpenSIGLE repository to explore possible unpublished grey literature. Search terms and strategy were developed by considering previous systematic reviews of AI in medical imaging found in the Cochrane Database of Systematic Reviews, National Institute of Health Research (NIHR) Health Technology Assessment (HTA) database, and the Database of Abstracts of Reviews of Effects (DARE). The search terms and strategy used for MEDLINE are shown in Table A1 (Appendix A); alterations were made to suit each electronic database. Once eligible studies were identified, the Science Citation Index was used to identify further studies which cited those found using the original search terms, and references were manually screened to identify any further studies that may have been missed. All studies were considered up until the date of the search: 25 March 2021.

### 2.2. Selection Criteria

Studies were included if (i) they evaluated CAD for prostate cancer detection or classification on MRI, (ii) CAD performance was compared to radiologist interpretation and against a histopathological reference standard, (iii) the evaluation patient cohort was treatment-naïve, and (iv) a full-text article was available. Studies were excluded if (i) MRI sequences other than T1-weighted imaging, T2-weighted imaging, diffusion-weighted imaging, or dynamic contrast-enhanced imaging were used, (ii) the comparator radiologist(s) did not have access to at least axial T2-weighted imaging and diffusion-weighted imaging with apparent diffusion coefficient map for reporting, and (iii) the patient cohort used for testing was less than thirty patients.

### 2.3. Data Extraction

Studies were initially screened by relevance of title and abstract; full texts of the remaining studies were read independently by the two reviewers. Studies that met the selection criteria were included; any disagreements between the two reviewers were solved by reaching a consensus or consulting a third expert reviewer if necessary.

Extracted data were categorized broadly into patient and study characteristics, radiologist and CAD system characteristics, and diagnostic performance. Sensitivity, specificity, and area under the receiver operating characteristic curve (AUC) were extracted at both per-lesion and per-patient levels, with 95% confidence intervals where available. Where multiple CAD systems were assessed in the same study, the results corresponding to highest performing system were considered. In studies where the requisite performance statistics were not reported, the performance statistics were calculated from the available data if possible, and attempts were made to contact authors if data were missing or unclear from their article.

### 2.4. Risk of Bias Assessment

In light of the lack of standardized and validated quality assessment tools for assessing studies concerning AI in medical imaging, we used an adapted version of the Quality Assessment of Diagnostic Accuracy Studies (QUADAS-2) tool with additional signaling questions from the preliminary QUADAS-C tool [15,16] and a published editorial outlining key considerations when assessing radiology research on AI [17]. The QUADAS-2 adapted tool and additional signaling questions are shown in the Supplementary Materials.

### 2.5. Data Synthesis

Due to substantial heterogeneities in CAD system applications, study designs, algorithms employed, patient cohorts used for evaluation, evaluation strategies, and performance metrics, it was decided that analysis would be by narrative synthesis rather than statistical pooling. Meta-analysis is not recommended for diagnostic test accuracy studies where the patient cohorts and test settings significantly differ between studies and would likely produce a biased result [18]. Publication bias was not assessed as there are no recommended methods for diagnostic accuracy studies [18].

## 3. Results

### 3.1. Literature Search

**Figure 1:**
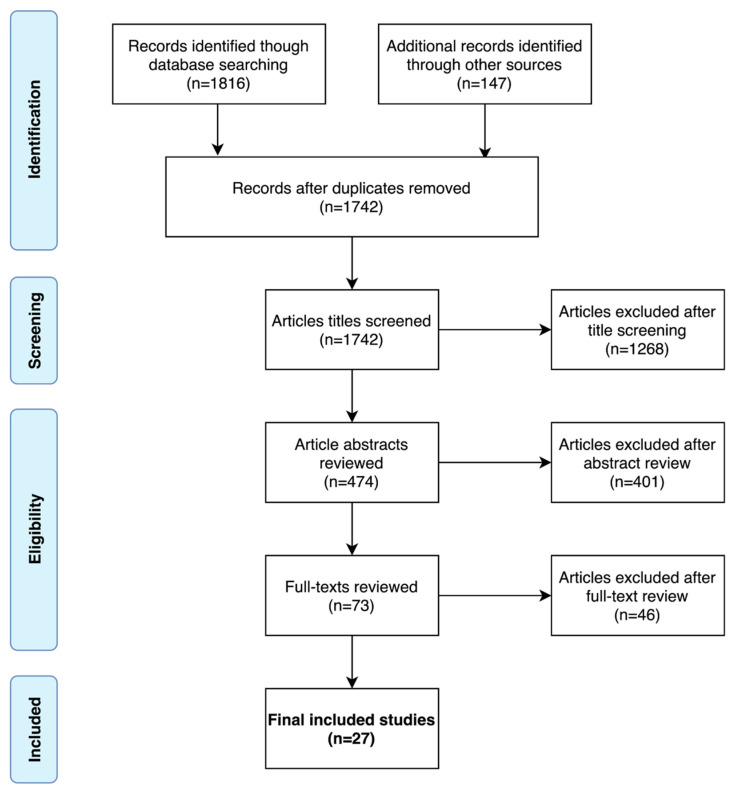
PRISMA flow diagram of the systematic search.

**Figure 2:**
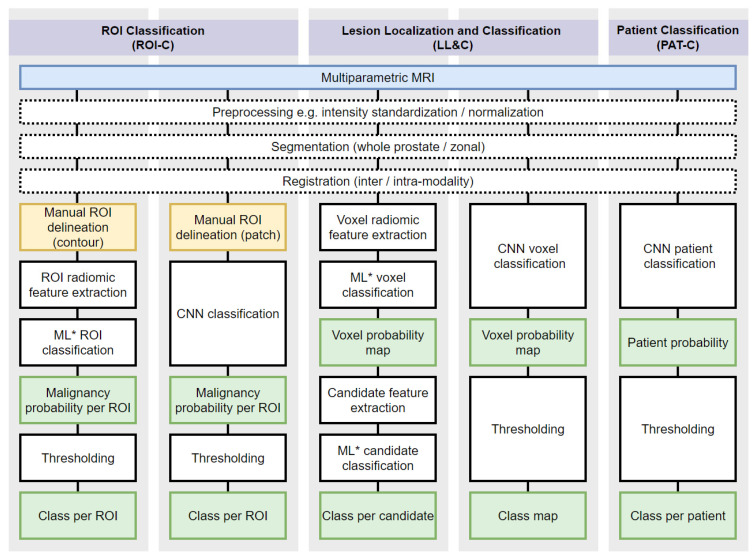
Workflow of typical prostate cancer AI CAD systems. Systems are categorized as ROI Classification (ROI-C), Lesion Localization and Classification (LL&C), or Patient Classification (PAT-C). Blue indicates mpMRI input, yellow indicates manual processes, white indicates automated processes, and green indicates intermediate or final outputs. ROI = region of interest. CNN = convolutional neural network. ML = machine learning. ML* here refers to ML algorithms exclusive of CNNs, such as support vector machines, random forest, logistic regression, and artificial neural networks.

A PRISMA flow diagram of the systematic search is shown in Figure 1. A total of 27 studies were included in the final analysis [19,20,21,22,23,24,25,26,27,28,29,30,31,32,33,34,35,36,37,38,39,40,41,42,43]. The 27 studies, and by extension, the CAD systems presented or evaluated within them, were categorized as either ROI Classification (ROI-C), Lesion Localization and Classification (LL&C), or Patient Classification (PAT-C); the categories are shown diagrammatically in Figure 2. ROI-C refers to (*n* = 16) studies where CAD systems classified pre-defined regions of interest (ROI), e.g., manually contoured lesions [19,20,21,22,23,24,25,26,27,28,29,30,31,32,44,45], LL&C refers to (*n* = 10) studies where CAD systems performed simultaneous lesion localization and classification [33,34,35,36,37,38,39,40,41,42], and PAT-C refers to (*n* = 1) studies where CAD systems classified patients directly [43].

### 3.2. Patient and Study Characteristics

Patient and study characteristics are summarized in Table 1. Studies were published between 2013 and 2021 from groups spanning Asia, Europe, and the USA. All 27 included studies used a retrospective study design. The median size of patient cohorts used for evaluation was 98 (range 30 to 417, *n* = 26) for studies where the size of the evaluation patient cohort was reported [19,20,21,22,23,24,25,26,27,28,29,31,32,33,34,35,36,37,38,39,40,41,42,43,44,45]. Most studies (*n* = 18) considered clinically suspected patient cohorts [20,21,22,23,27,31,34,35,36,37,38,39,40,41,42,43,44,45], while fewer studies (*n* = 9) considered patient cohorts with biopsy-proven prostate cancer [19,24,25,26,28,29,30,32,33]. Histopathological reference standards used in studies were one or a combination of the following: transperineal template prostate-mapping (TTPM) biopsy, in-bore targeted biopsy, TRUS targeted biopsy, TRUS saturation biopsy, TRUS systematic biopsy, or radical prostatectomy. The majority of studies (*n* = 22) collected scans using 3T MR scanners [19,20,21,23,24,25,29,30,31,32,33,34,36,37,38,39,40,41,42,43,44,45], while fewer studies (*n* = 4) used 1.5T MR scanners [22,26,27,35]; one further study used 3T mainly but included one scan acquired at 1.5T [28]. Evaluation using multicenter data was completed in three studies [19,34,38], and only three studies used multivendor MRI data for evaluation [19,23,34].

### 3.3. Reader and CAD System Characteristics

Reader and CAD system characteristics are summarized in Table 2. In the majority of studies (*n* = 26), readers scored suspicious lesions using mpMRI [20,21,22,23,24,25,26,27,28,29,30,31,32,33,34,35,36,37,38,39,40,41,42,43,44,45], while in one study, biparametric MRI (bpMRI) was used [19]. In 15 studies, patient cases were reported by a single reader [19,20,21,22,23,28,32,33,37,38,39,40,42,43,44], while in the remaining 12 studies, patient cases were reported by more than one reader [24,25,26,27,29,30,31,34,35,36,41,45]; where multiple readers reported on each patient, the presented reader performance is either an average of reader performance or based on the consensus view of readers. Reader experience varied significantly across studies; in studies where reader performance was stratified by experience level, details of the most experienced reader group were extracted. Considerable heterogeneity was observed in the machine learning algorithms employed by the CAD systems: five studies presented convolutional neural networks (CNN) [32,33,39,40,43], one study evaluated the commercially available Watson Elementary^TM^ system [41], while the remaining 21 studies presented or evaluated CAD systems based on traditional machine learning algorithms [19,20,21,22,24,25,35,36,37,38,41,45]. Across the studies, a variety of methods were used to construct datasets for training and evaluation. Six studies used random splitting [19,30,31,32,39,43], five studies used temporal splitting [21,22,23,40,45], five studies used leave-one-patient-out (LOPO) cross-validation [24,25,26,29,37], four studies used an independent internal testing cohort [35,36,42,44], four studies obtained external data for testing [28,34,38,41], two studies used five-fold cross-validation [20,33], and one study did not report how they separated data for training and evaluation [27].

### 3.4. Risk of Bias Assessment

**Figure 3:**
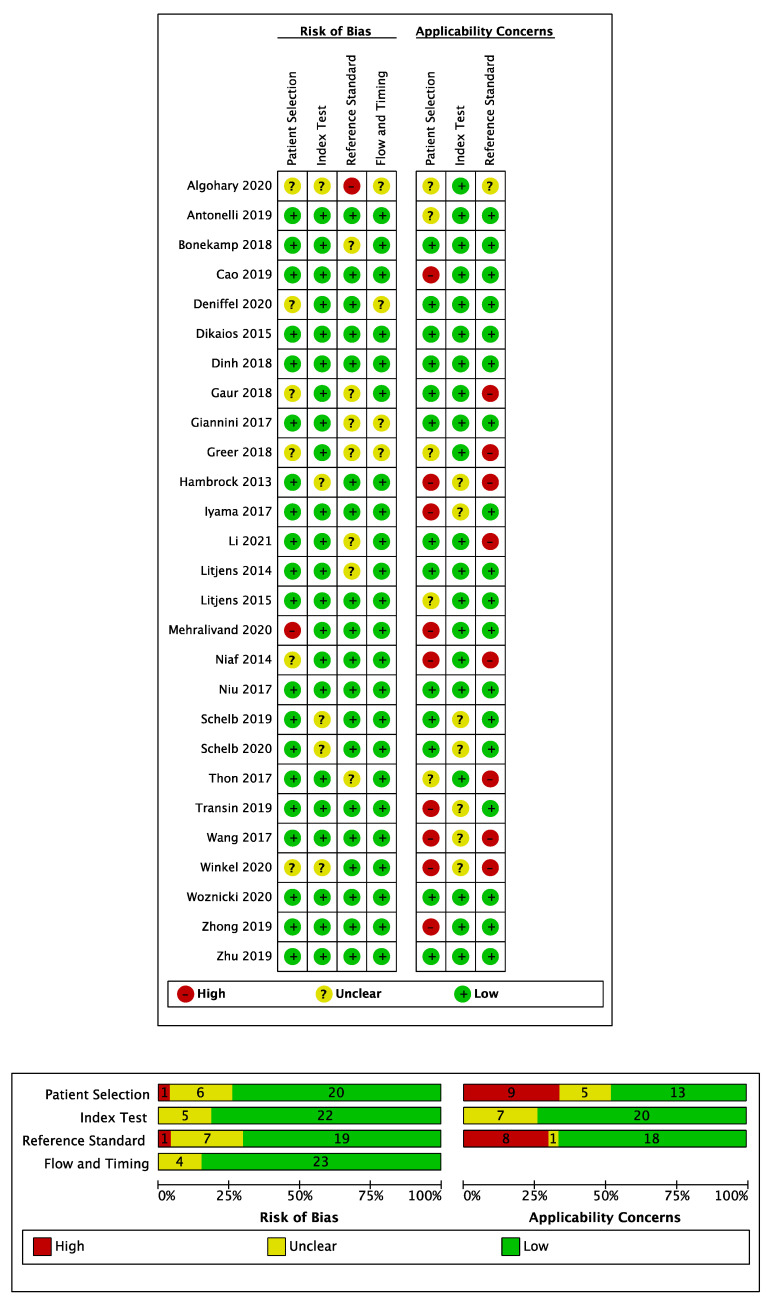
QUADAS-2 risk of bias and applicability concerns summary for all included studies.

A summary of the QUADAS-2 assessment of included studies is shown in Figure 3. Generally, a low risk of bias was observed for patient selection. The majority of studies (*n* = 20) included consecutive patient cohorts with appropriate inclusion and exclusion criteria [20,21,22,23,24,25,27,28,29,31,32,33,35,37,39,40,41,42,44,45]. However, in six studies the risk of bias for patient selection was unclear due to an insufficient description of case selection [19,26,30,34,36,43], and one study had a high risk of bias for patient selection due to a case–control design [38]. There was a high concern over the applicability of patient cohorts in eight studies featuring biopsy-proven patient cohorts, where a radical prostatectomy reference standard was used [24,25,26,28,29,32,33,38], due to the spectrum bias associated with patients who undergo radical prostatectomy [46] and a lack of cases without prostate cancer. In addition, one further study that only included patients who underwent radical prostatectomy or had a negative mpMRI, and therefore lacked representation of benign false positive MR findings, was also deemed to have a high applicability concern [38]. Patient applicability was unclear in three studies where men were imaged following an initial negative TRUS biopsy only, which differs to the modern-day pre-biopsy setting of MRI [20,41,44], and in one study where baseline demographics were omitted [19]. In the remaining studies, concerns over patient cohort applicability were deemed low [21,22,23,27,31,34,35,36,37,39,40,42,43,45]. The risk of bias for the index test was low in all but six studies where it was deemed high due to the lack of a pre-specified model cut-off value for calculating sensitivity and specificity [19,30] or due to determination of the model cut-off value using the same test set data to which the cut-off was subsequently applied, which likely overestimates the performance that would be attained during prospective use [29,32,41]. The risk of bias for the index test was unclear in one study when the radiologist was aware all cases contained cancer [24]. Concerns over the applicability of the index test were generally low, however, applicability was unclear in four studies where post-biopsy MRI examinations were considered [24,25,28,29] and one study that featured a mixed-use of PI-RADS v1 and v2 scoring systems [39], neither of which are common in current clinical practice.

The risk of bias was low for the reference standard used in 21 studies. However, one study had a high risk of bias for the reference standard, which was 12-core systematic TRUS biopsy without supplementation by other biopsy types [19], which is known to miss clinically significant disease [6]. In the remaining five studies, the risk of bias was unclear as TRUS biopsy was used to determine negative cases [34,36,38,45] or due to the lack of histopathological follow-up of some MR negative cases [37]. Concerns over the applicability of the study endpoint as defined by the reference standard were high in eight studies [24,26,29,30,34,36,41,45], where the study endpoint did not include the condition Gleason score ≥ 3 + 4; endpoints which did not include this condition were marked with a high applicability concern due to their misalignment with generally accepted definitions of clinically significant cancer [47]. 

All 27 studies were deemed to have a low risk of bias for study flow and timing, with consistent use of reference standards for included patients and appropriate intervals between MRI and obtaining histopathology.

A further quality assessment was conducted against the key considerations for authors, reviewers, and readers of AI Manuscripts in radiology by Bluemke et al. [17]; a summary of the quality assessment is shown in Table 3. The vast majority of studies adequately defined image sets, used widely accepted reference standards for training, and described the preparation of images. However, the remaining key considerations were only addressed by small subsets of the included studies: only four studies used external test sets for final statistical reporting [28,34,38,41], only four studies used multivendor images for evaluation [19,23,34,38], only three studies justified dataset sizes using statistical sample size calculations [34,36,38], only six out of 16 ROI-C studies (and all LL&C and PAT-C studies) demonstrated how the AI algorithm makes decisions by reporting a model cut-off value, and only three studies featured publicly available systems [39,40,41].

### 3.5. ROI Classification Summary of Results

Per-lesion performance for all 16 ROI-C studies is presented in Table 4. Three ROI-C studies further reported per-patient performance, which is presented in Table 5. The majority of ROI-C studies (*n* = 15) presented CAD systems based on traditional non-deep learning machine learning algorithms with radiomic and clinical feature input [19,20,21,22,23,24,25,26,27,28,29,30,31,45], while one study used a CNN to classify lesion-centered patches [32]. 

Of the 16 ROI-C studies, 11 reported standalone CAD performance, where the output was thresholded to give a sensitivity and specificity [19,20,21,22,23,27,28,29,30,32,45]. Of those 11 studies, three reported superior diagnostic accuracies for CAD compared to the radiologist, with statistical significance, either by sensitivity [29], specificity [23], or both [27]. In contrast, one study showed inferior sensitivity for CAD compared to the radiologist, with statistical significance [28]; among ROI-C studies, only this study performed an evaluation using externally obtained test data. The remaining seven studies showed no significant differences between CAD and radiologists in either sensitivity or specificity [19,20,21,22,30,32,45]. Methods used to threshold the output of CAD systems were reported in seven of the 11 studies [20,21,22,23,28,29,32]. Five studies avoided bias by not using the test cohort when picking the cut-off value [20,21,22,23,28], while in two studies, the cut-off value was chosen using Youden statistics [29] or the point of maximum accuracy [32] on the test cohort. Three studies reported a lesion-level AUC only rather than thresholding their CAD systems’ output, with one study reporting a significantly higher AUC than readers [25] and two studies reporting no significant difference [24,26].

An ensembled CAD system incorporating the radiologist’s reporting score was investigated in four studies [29,31,44,45], three of which showed significant improvement upon the radiologist’s score alone. Li et al. [45] combined a CAD likelihood score with a PI-RADS v2.1 score and a prostate-specific antigen (PSA) value, using a logistic regression classifier, reporting an increased AUC compared to radiologist PI-RADS v2.1 assessment alone, with statistical significance. In Litjens et al. [44], a CAD likelihood score was combined with a PI-RADS v1 score, using a logistic regression classifier; they reported an increased specificity over radiologist assessment using PI-RADS v1 alone, with statistical significance. In Wang et al. [29], a support vector machine classifier was used to combine radiomic features and a PI-RADS v2 score; they found an increase in sensitivity over radiologist PI-RADS v2 assessment alone, with statistical significance. A further two studies compared radiologist interpretation with and without knowledge of CAD scores [24,26], for which no significant differences were demonstrated.

### 3.6. Lesion Localization and Classification Summary of Results

Ten studies investigated the use of CAD systems for simultaneously localizing and classifying lesions. Table 4 and Table 5 show per-lesion and per-patient results, respectively. Six studies evaluated traditional non-deep learning machine learning algorithms [34,35,36,37,38,42], three studies evaluated CNNs [33,39,40], and one study evaluated the commercially available Watson Elementary^TM^ system [41]. 

Five studies’ primary objective was to investigate the standalone performance of CAD systems for localizing and classifying lesions [33,37,39,40,41]. Of these, only the studies presented by Schelb et al. [39,40] reported sensitivity and specificity by choosing a cut-off determined without using test data. Neither study reported a statistically significant difference in sensitivity or specificity between CAD and readers at both per-lesion and per-patient level, on internal test cohorts. 

Five studies investigated the role of CAD systems in assisting readers to localize and classify suspicious lesions [34,35,36,38,42]. In four of those studies, readers could only approve or reject lesions highlighted by the CAD system’s output voxel probability map [34,35,36,38]. Gaur et al. [34] evaluated this paradigm on a multicenter external test cohort featuring scans from five institutions based in four countries; they found that CAD assistance significantly lowered the per-patient sensitivity and increased the per-patient specificity compared to readers alone. Similarly, Mehralivand et al. [38] evaluated CAD-assistance using a multicenter external test cohort collected from five institutions; they found that CAD-assistance did not significantly improve per-patient sensitivity, while specificity was not presented. In the other similar studies where readers were confined to accept or reject CAD highlighted areas [35,36], one study showed an improved per-patient sensitivity for CAD-assistance on an independent internal test cohort, with statistical significance [35], and one study showed a reduced per-patient specificity for CAD-assistance on an independent internal test cohort, with statistical significance [36]. Rather than restrict readers to choose from CAD highlighted areas only, Zhu et al. [42] compared the unconstrained performance of readers before and after seeing the CAD system’s output; they found that CAD-assisted diagnosis increased per-patient sensitivity, with statistical significance, compared to readers alone, on an independent internal test cohort.

### 3.7. Patient Classification Summary of Results

The study by Deniffel et al. [43] was the only PAT-C study that met the selection criteria. Their presented CAD system directly classified patients into those with and without clinically significant cancer using a CNN classifier. At probability threshold ≥ 0.2, CAD system per-patient sensitivity and specificity exceeded that of readers. However, since the threshold was not pre-specified or determined using training data, the performance may not be a true reflection of how the classifier would perform prospectively.

## 4. Discussion

This systematic review highlights the extensive efforts of research groups globally who are seeking to address known issues in the prostate cancer diagnostic pathway through the introduction of AI technologies. A combination of clinicians and algorithm developers worked on all aspects of this systematic review to ensure accurate analysis and sufficient critique of the information presented in the studies. Twenty-seven studies were included in the final analysis. Studies were categorized as ROI-C, LL&C, and PAT-C. The key selection criteria for inclusion was reported radiologist performance to which the performance of CAD systems could be compared.

Among the 16 ROI-C studies, the study by Dinh et al. [23] was of a particularly high quality based on its QUADAS-2 assessment. The generalized linear mixed model classifier-based CAD system they presented showed superior performance compared to radiologist Likert scoring on a consecutive patient cohort of size 129 with combined systematic and targeted biopsy reference standard. A high sensitivity cut-off value was considered for both the CAD system and radiologist to minimize missed clinically significant cancers; radiologist Likert scoring was thresholded using cutoff ≥3, while the CAD system was thresholded using a cut-off value corresponding to 95% sensitivity in the training set. A per-patient sensitivity of 100% (95% CI: 100–100%) and specificity of 9% (95% CI: 2–15%) was reported for radiologist Likert scoring, while a per-patient sensitivity of 100% (95% CI: 100–100%) and specificity of 40% (95% CI: 28–51%) was reported for the CAD system. Therefore, CAD system use would result in 31% less unnecessary biopsies, while ensuring no patients with clinically significant prostate cancer are missed. However, their performance comparison considered an internal test set only. Conversely, the study by Transin et al. [28] was the only ROI-C study to use an external test set; they evaluated the same CAD system as Dinh et al., but found CAD system sensitivity to be 89% (95% CI: 82–97%) which was significantly lower than the radiologist sensitivity of 97% (95% CI: 93–100%), without an improvement in specificity.

Among LL&C studies, the study by Zhu et al. [42] was high quality as reflected by its QUADAS-2 assessment. Further to this, we believe the CAD-assistance paradigm evaluated in their study is the most likely to be clinically translatable. In their study, readers were permitted to score all lesions, including those not highlighted by their artificial neural network classifier-based CAD system. Per-patient sensitivity increased from 84% (95% CI: 75–91%) unassisted, at PI-RADS v2 threshold ≥3, to 93% (95% CI: 86–98%) CAD-assisted and specificity increased from 56% (95% CI: 43–69%) to 66% (95% CI: 53–77%), on an independent internal test cohort of size 153. It should be noted that their study considered CAD-assistance for relatively inexperienced radiologists (1–2 years), where the impact of CAD-assistance may be the greatest. The studies by Gaur et al. [34] and Mehralivand et al. [38] must also be highlighted; both studies evaluated CAD using images acquired from five centers based across multiple countries. Such studies have a large role to play in providing supporting evidence for the clinical translation of CAD systems. These studies reported similar diagnostic accuracy between radiologists with and without CAD assistance, on patient cohorts of size 216 and 236, respectively, indicating the potential for widely generalizable systems that can be clinically deployed.

Due to the marked heterogeneity in study designs, algorithms employed, datasets evaluated upon, evaluation strategies, and performance metrics, it was not possible to perform a meta-analysis or to draw conclusions on whether any particular class of algorithms outperformed others. Furthermore, deficiencies in the included studies meant we could not conclude the readiness of any presented CAD system to be deployed clinically. We now provide recommendations for future studies.

Firstly, CAD evaluation studies and underlying algorithms should be designed with a clinically relevant question or use in mind. A specific use of CAD within the diagnostic pathway will mandate the ideal characteristics of the patient cohort and reference standard of both training and test sets and inform the appropriate thresholding and benchmark for performance outcomes. The majority of studies included in this systematic review did not indicate their intended use a priori. For ROI-C systems, it seems appropriate that CAD could be used to further inform the decision to biopsy following lesion detection by a radiologist. In this setting, a desirable CAD system would maintain high sensitivity to minimize missed cancers, while improving the specificity of radiologist scoring to reduce unnecessary biopsies, particularly for indeterminate lesions where the rate of clinically significant cancer on biopsy is only 13–21% [7]. In comparison, LL&C systems may be used by radiologists concurrently during reporting to highlight suspicious areas with the hope of improving detection sensitivity. LL&C systems such as those presented in this review, which matched the sensitivity of expert radiologists, can improve the sensitivity of less experienced radiologists, and reduce missed cancers due to human error, distraction, or fatigue. Alternatively, PAT-C may have a role to play as a first reader to either prioritize examinations for radiologists to report or to identify negative cases that may not need radiologist review at all. The intended use of the CAD system should be reflected in the evaluation setting, and although knowledge of the stand-alone performance of CAD systems may be helpful in providing context and confidence to radiologists in their underlying performance, assessment of the interaction between radiologists and the CAD system should be made in line with the CAD system’s planned clinical use. Moreover, we note that in Giannini et al., readers had variable changes in their diagnostic accuracy when using CAD, likely reflecting individual readers’ trust in the CAD system versus their own experience. Therefore, multiple-reader studies are preferred [35].

Secondly, test sets should be appropriate and well-curated in terms of size, diversity, and relevance. Many included studies used small patient cohorts for evaluation, irrespective of evaluation strategy. The largest evaluation cohort among the included studies was in the study by Cao et al. [33], where five-fold cross-validation was applied using 417 patients with 728 lesions, 442 of which were Gleason score ≥3 + 4. Studies should determine the minimum sample size required to adequately power a study to detect some clinically relevant effect size for CAD and to allow statistically valid comparisons [48]; among the studies included in this review, only Gaur et al. [34], Greer et al. [36], and Mehralivand et al. [38] included such calculations. Notably, the majority of included studies used cross-validation of internal evaluation cohorts from a single center and MR scanner, which prohibit understanding of the generalizability of the CAD system. Held-out test sets completely independent of the training set are preferred to cross-validation/internal validation, and should include diverse data from multiple centers and MR vendors, as in Gaur et al. [34]. We note that those studies using external test cohorts did not demonstrate the superior performance of CAD versus radiologists [28,34,38,41], as seen in some studies using internal datasets [23,35,42]. The likely cause for less optimistic results of CAD evaluated using external test cohorts is a generalization gap due to the varying appearances of MRI obtained from scanners with different manufacturers, field strengths, and acquisition parameters. It would be interesting to study the specific differences which cause the largest generalization gaps, and present results for individual scanners in future work. In addition, calibration of CAD systems to external MR data should also be considered to improve performance on external test cohorts.

Thirdly, CAD evaluation studies should use a widely accepted and accurate histopathological reference standard. For biopsy naïve populations, a reference standard that combines targeted biopsy with a biopsy technique that samples the gland frequently and systematically, such as transperineal template prostate-mapping (TTPM) biopsy, is favored over prostatectomy, due to the associated spectrum bias. However, few studies used a TTPM biopsy reference standard as it is usually reserved for planned clinical trials, suggesting the need for specific planned clinical trials for CAD system evaluation, as opposed to the current practice of evaluating CAD systems using retrospective clinical data. In addition, care should be taken when using MR-negative cases without histopathological confirmation for CAD system training and evaluation. It is important to avoid discarding such cases if MR-negative cases with histopathological confirmation are not available, to avoid a spectrum bias towards radiologically abnormal MRIs; in these cases, long-term follow-up or expert consensus reviews may be sufficient as a reference standard.

Fourthly, CAD evaluation studies should consider non-imaging data sources. Remarkably, only two studies used clinical data outside of the imaging and radiologist score [31,45]. Although the focus is often on the available MR data, non-imaging biomarkers such as PSA density have been shown to be useful predictors of clinically significant cancer; incorporating such data when available, alongside MR data, may enhance algorithms [49,50].

Fifthly, the choice of performance measures used to evaluate CAD systems should be pre-specified and hold appropriate clinical context for comparison to radiologists. Regrettably, some studies only reported an AUC, and others introduced bias by thresholding the probabilistic output of their CAD systems using the test cohort or not specifying how thresholds were chosen. The output of CAD systems should be thresholded without knowledge of the test set, to produce an unbiased measure of sensitivity and specificity. The choice of operating point will depend on the accepted risk threshold for a particular population. However, logical clinical reasoning should be applied to achieve a desired sensitivity or specificity for the particular use case. Alternative statistical methods such as decision curve analysis, used by Deniffel et al., may be appropriate if authors wish to compare across a range of risk thresholds. Thresholds for LL&C algorithms may be best chosen by the acceptable false-positive rate that still delivers a sufficiently high sensitivity for clinically significant cancer. High false-positive rates were noted in multiple studies [34,35,39], and efforts to quantify an acceptable false-positive rate for prostate mpMRI CAD may be helpful, as has been done in other applications of CAD [51].

Sixthly, more CAD systems must be made publicly available, to allow the most promising CAD systems to be evaluated more extensively by the community. Among the CAD systems presented and/or evaluated in this systematic review, only the deep learning CAD system presented and evaluated in the studies by Schelb et al. [39,40] and the Watson Elementary^TM^ system evaluated in the study by Thon et al. [41] have been made publicly available. Alternatively, curation and sharing of large, diverse, and well-labelled datasets would allow direct comparisons of algorithms and potentially expedite the development of more robust and generalizable CAD systems. Thankfully efforts are underway for sharing prostate imaging data between centers and commercial companies, and furthermore, well-designed AI challenges in prostate MR may be a solution to evaluate and compare multiple algorithms externally [52,53].

Finally, prospective evaluation of CAD systems is necessary to simulate clinical deployments and avoid biases that can affect retrospective evaluation. In this systematic review, we were not able to identify any prospective evaluation studies that met our selection criteria. For impactful prospective evaluation, consideration about how the CAD output is presented to clinicians and used within the diagnostic pathway is crucial. Notably, Schelb et al. [40] simulated clinical deployment of their CAD system with retrospective data and highlighted the considerations needed for ongoing quality assurance to maintain and optimize performance over time at a single center; their study is a useful and practical step towards true prospective evaluation.

There are some limitations to this review. Firstly, whilst we believe our search strategy was comprehensive, there is a possibility that some relevant studies may not have been included, in particular those studies that may have been published in the time between our search and publication of this review. Secondly, the heterogeneity of studies dictated our choice of narrative synthesis rather than meta-analysis, restricting direct comparisons between study outcomes and proclamation of the superiority of particular algorithms or a class of algorithms. In particular, the variability of individual studies’ definitions of clinically significant cancer, which are likely to have had a large impact on reported radiologist and CAD performance, was a major factor in our decision not to conduct a meta-analysis or to compare studies directly. Finally, this systematic review focused on diagnostic accuracy and did not discuss other important outcomes for CAD such as improvements in reporting time or inter-reader agreement.

## 5. Conclusions

In conclusion, we found a lack of evidence to support the deployment of CAD systems based on AI algorithms for the initial diagnosis of prostate cancer on MRI, presently. Of the studies that met the selection criteria for this systematic review, none followed a prospective study design, and a performance benefit from CAD was only seen in studies that performed a retrospective evaluation using internal patient datasets. In the few studies that evaluated CAD using externally obtained patient data, CAD performance was either inferior to or on-par with radiologists alone. Future studies must show a performance benefit from CAD prospectively in external, multicenter settings, and must avoid the methodological flaws identified in the studies included in this systematic review. In addition, future studies must be designed to answer clinically relevant questions and describe the specific clinical use of the CAD system they present. Greater efforts by the community to build bespoke, high-quality large public datasets to enable the robust external and prospective evaluation of CAD required, will accelerate progress substantially.

## Figures and Tables

**Table 1:** Patient and Study Characteristics.

Study	Year	Country	No. of Patients	Age, Years	PSA, ng/mL	Patient Population	Pre- or Post- Biopsy MRI	Field Strength, T	*n*° Centers/Vendors	Reference Standard
ROI Classification (ROI-C)										
Algohary [19]	2020	USA	115	NR	NR	Biopsy proven	Pre-biopsy	3	4/2	TRUS (12-core)
Antonelli [20]	2019	UK	134	64 (43–83)	7.4 (2.5–30.3)	Clinically suspected	Pre-biopsy	3	1/1	TTMB +/− TB
Bonekamp [21]	2018	Germany	133	63 (58–71)	7.5 (5.4–11)	Clinically suspected	Pre-biopsy	3	1/1	TB
Dikaios [22]	2015	UK	85	63 * (45–77)	8.7 * (0.2–39)	Clinically suspected	Pre-biopsy	1.5	1/1	TTMB
Dinh [23]	2018	France	129	67 (IQR 61–70)	7.3 (IQR 5.1–11.5)	Clinically suspected	Pre-biopsy	3	1/2	TRUS (4-core) + TB
Hambrock [24]	2013	Netherlands	34	64 * (53–74)	7.5 * (3.4–21.8)	Biopsy proven	Post-biopsy	3	1/1	RP
Iyama [25]	2017	Japan	60	70 * (55–81)	10.4 * (5–160)	Biopsy proven	Unclear	3	1/1	RP
Li [45]	2021	China	62	65 * (SD 9.4)	NR	Clinically suspected	Pre-biopsy	3	1/1	TRUS (12-core)/RP
Litjens [44]	2015	Netherlands	107	66 (48–83)	13 (1–56)	Clinically suspected ^‡^	Pre-biopsy	3	1/1	TB
Niaf [26]	2014	France	30	62 (45–70)	7.4 (4.6–40)	Biopsy proven	Post-biopsy	1.5	1/1	RP
Niu [27]	2017	China	184	59 (42–79)	12.0 (4–98.3)	Clinically suspected	Pre-biopsy	1.5	1/1	TRUS (12-core) + TB
Transin [28]	2019	France	74	66 (IQR, 62–69)	7.4 (IQR 5.7–11)	Biopsy proven	Post-biopsy	3/1.5 ^§^	1/1	RP
Wang [29]	2017	China	54	74 (IQR, 66–78)	23.6 (IQR, 12.5–56.1)	Biopsy proven	Post-biopsy	3	1/1	RP
Winkel [30]	2020	Switzerland	40 **	69 * (SD 8.7) ^†^	7 * (SD 11.2) ^†^	Biopsy proven	Pre-biopsy	3	1/1	TRUS (12/18 core) + TB
Woźnicki [31]	2020	Germany	40	69 (IQR 63–72)	8.2 (IQR 6.8–11.9)	Clinically suspected	Pre-biopsy	3	1/1	TRUS (12-core) + TB
Zhong [32]	2019	USA	30	NR (43–80) ^†^	7.9 * (SD 12.5) ^†^	Biopsy proven	Mix	3	1/1	RP
Lesion Localization and Classification (LL&C)										
Cao [33]	2019	USA	417	NR	NR	Biopsy proven	Pre-biopsy	3	1/1	RP
Gaur [34]	2018	USA	216	62 * (42–79)	8.1 * (0.3–31.9)	Clinically suspected	Pre-biopsy	3	5/3	RP/TRUS (12/24-core)
Giannini [35]	2017	Italy	89	67 (63–73)	7.5 (6.2–11.0)	Clinically suspected	Pre-biopsy	1.5	1/1	TB/Saturation biopsy ^^^
Greer [36]	2018	USA	163	62 * (43–83)	9.8 * (1.5–84.6)	Clinically suspected	Pre-biopsy	3	1/1	RP/TRUS (12-core)
Litjens [37]	2014	Netherlands	347	NR	NR	Clinically suspected	Pre-biopsy	3	1/1	TB
Mehralivand [38]	2020	Multiple	236	NR	NR	Clinically suspected	Pre-biopsy	3	5/NR	RP/TRUS (12-core)
Schelb [39]	2019	Germany	62	64 (IQR 60–69)	6.9 (IQR 5.1–8.9)	Clinically suspected	Pre-biopsy	3	1/1	TRUS + TB
Schelb [40]	2020	Germany	259	61 (IQR 61–72)	7.2 (IQR 5.2–10.0)	Clinically suspected	Pre-biopsy	3	1/1	TB + Saturation biopsy
Thon [41]	2017	Germany	79	65 * (48–80)	NR	Clinically suspected ^‡^	Pre-biopsy	3	1/1	TB
Zhu [42]	2019	China	153	66 (IQR 30–73)	12.3 (IQR 7.9–21)	Clinically suspected	Pre-biopsy	3	1/1	TRUS (12/13-core) + TB
Patient Classification (PAT-C)										
Deniffel [43]	2020	Canada	50	64 * (SD 8.4)	7.2 (IQR 5.2–11.2)	Clinically suspected	Pre-biopsy	3	1/1	TB +/− TRUS (12-core)

(Age and PSA median (min-max range) reported unless stated otherwise; IQR—interquartile range; MRI—magnetic resonance imaging; NA—not applicable; NR—not reported; PSA—prostate-specific antigen; RP—radical prostatectomy; SD—standard deviation; T—tesla; TB—targeted biopsy; TTMB—transperineal template prostate-mapping biopsy; TRUS—transrectal ultrasound-guided biopsy)* Mean † Includes training and test set ‡ Previous negative systematic biopsy § Only one scan using 1.5T ^ PSA and MRI surveillance were used in patients with negative mpMRI who did not undergo biopsy ** Lesions reported.

**Table 2:** Reader and CAD System Characteristics.

Study	Reader Characteristics			CAD System Characteristics			
	MRI	No. of Readers ^§^	Reader Experience, Years	Algorithm	Input Sequences	Discriminative Features	Evaluation Strategy
ROI Classification (ROI-C)							
Algohary [19]	bp	1	7–25	QDA	T2WI, ADC	ROI radiomic features (intra-tumoral and peri-tumoral)	Randomly split test cohort
Antonelli [20]	mp	1	10 (>100 MRIs/year)	PZ: LinR, TZ: NB	ADC, DCE, PSAd	ROI radiomic features, PSAd	Five-fold CV
Bonekamp [21]	mp	1	>3 *	RF	T2WI, ADC, DWI (b1500)	ROI radiomic features	Temporally split test cohort
Dikaios [22]	mp	1	7 (300–400 MRIs/year)	LogR	T2WI, ADC, DCE	ROI radiomic features	Temporally split test cohort
Dinh [23]	mp	1	0.25–17	GLMM	ADC, DCE	ROI radiomic features	Temporally split test cohort
Hambrock [24]	mp	4 ^‡^	NR (>100 MRIs)	LDA	ADC, DCE	ROI radiomic features	LOPO CV
Iyama [25]	mp	2	7	LogR	T2WI, ADC	ROI radiomic features	LOPO CV
Li [45]	mp	2	>5	LogR	T2WI, ADC, DWI (b1000), DCE	ROI radiomic features, PI-RADS v2.1 score, PSA	Temporally split test cohort
Litjens [44]	mp	1	2–20	LogR	PDI, T2WI, ADC, DWI (b800), DCE	RF likelihood, PI-RADS v1 score	Internal independent test cohort
Niaf [26]	mp	5 ^‡^	1–7	SVM	T2WI, DWI (b600), DCE	ROI radiomic features	LOPO CV
Niu [27]	mp	2	1–4	LogR	T2WI, ADC	ROI radiomic features	NR
Transin [28]	mp	1	20	GLMM	ADC, DCE	ROI radiomic features	External test cohort
Wang [29]	mp	2	>10	SVM	T2WI, ADC, DWI (b1500), DCE	ROI radiomic features, PI-RADS v2 score	LOPO CV
Winkel [30]	mp	2	>5	RF	T2WI, ADC, DCE	ROI radiomic features	Randomly split test cohort
Woźnicki [31]	mp	2	>7	Ensemble (SVM, LogR)	T2WI, ADC	ROI/WG radiomic features, PI-RADS v2 score, PSAd, DRE findings	Randomly split test cohort
Zhong [32]	mp	1	>10 (>500 MRIs/year)	CNN	T2WI, ADC	CNN learned features	Randomly split test cohort
Lesion Localization and Classification (LL&C)							
Cao [33]	mp	1	>10 (>1000 MRIs/year)	CNN	T2WI, ADC	CNN learned features	Five-fold CV
Gaur [34]	mp	2	NR (500–2000 MRIs/year)	RF	T2WI, ADC, DWI (b1500)	Voxel radiomic features	Multicenter external test cohort
Giannini [35]	mp	3	2–4 (120–200 MRIs/year)	SVM	T2WI, ADC, DCE	Voxel radiomic features	Internal independent test cohort
Greer [36]	mp	2	NR (<500–2000 MRIs/year)	RF	T2WI, ADC, DWI (b2000)	Voxel radiomic features	Internal independent test cohort
Litjens [37]	mp	1	20 ^†^	RF	PDI, T2WI, ADC, DWI (b800), DCE	Stage 1: voxel radiomic features, Stage 2: candidate radiomic features	LOPO CV
Mehralivand [38]	mp	1	<1–>3 or <100–>300 MRIs/year	RF	T2WI, ADC, DWI (b1500)	Patch-based radiomic features	Multicenter external test cohort
Schelb [39]	mp	1	>3 *	CNN	T2WI, ADC, DWI (b1500)	CNN learned features	Randomly split test cohort
Schelb [40]	mp	1	>3 *	CNN	T2WI, ADC, DWI (b1500)	CNN learned features	Temporally split test cohort
Thon [41]	mp	2	NR	Watson Elementary^TM^	T2WI, ADC, DCE	Voxel radiomic features	External test cohort
Zhu [42]	mp	1	1–2 (200 MRIs/year)	ANN	T2WI, ADC, DCE	Voxel radiomic features	Internal independent test cohort
Patient Classification (PAT-C)							
Deniffel [43]	mp	1	3–15	CNN	T2WI, ADC, DWI (b1600)	CNN learned features	Randomly split test cohort

(ADC—apparent diffusion coefficient; ANN—artificial neural network; b—b-value; bp—biparametric; CAD—computer-aided diagnosis; CNN—convolutional neural network; CV—cross-validation; DCE—dynamic contrast-enhanced imaging; DWI—diffusion-weighted imaging; GLMM—generalized linear mixed model; LinR—linear regression; LogR—logistic regression; LOPO—leave-one-patient-out; mp—multiparametric; MRI—magnetic resonance imaging; NB—naïve Bayes; NR—not reported; PDI—proton density image; PI-RADS—Prostate Imaging-Reporting and Data System; PSAd—prostate specific antigen density; PUN—phenomenological universalities; PZ—peripheral zone; QDA—quadratic discriminant analysis; RF—random forest; ROI—region of interest; SVM—support vector machine; T2WI—T2-weighted imaging; TZ—transition zone; WG—whole gland). * One radiologist with less than 3 years of experience reported 2% of examinations. † Reported by or under the supervision of an expert radiologist (>20 years). ‡ Inexperienced readers not included. § Minimum readers per scan.

**Table 3:** Summary of Key Considerations for Artificial Intelligence Studies in Radiology.

Study	Are All Applicable Image Sets Defined?	Is an External Test Set Used for Final Statistical Reporting?	Have Multivendor Images Been Used to Evaluate the AI Algorithm?	Are the Size of the Training, Validation and Test Sets Justified?	Was the Algorithm Trained Using a Standard of Reference That Is Widely Accepted in the Field?	Was the Preparation of Images for the AI Algorithm Adequately Described?	Were the Results of the AI Algorithm Compared with Expert Radiologists?	Was the Manner in Which the AI Algorithm Makes Decisions Demonstrated?	Is the AI Algorithm Publicly Available?
ROI Classification (ROI-C)									
Algohary [19]	✗	✗	✓	✗	✗	✓	✓	✗	✗
Antonelli [20]	✓	✗	✗	✗	✓	✓	✓	✓	✗
Bonekamp [21]	✓	✗	✗	✗	✗	✓	✓	✓	✗
Dikaios [22]	✓	✗	✗	✗	✓	✓	✓	✓	✗
Dinh [23]	✓	✗	✓	✗	✓	✓	✓	✓	✗
Hambrock [24]	✓	✗	✗	✗	✓	✓	✓	✓	✗
Iyama [25]	✓	✗	✗	✗	✓	✓	✓	✗	✗
Li [45]	✓	✗	✗	✗	?	✓	✓	✗	✗
Litjens [44]	✓	✗	✗	✗	✓	✓	✓	?	✗
Niaf [26]	✓	✗	✗	✗	✓	✗	✓	✗	✗
Niu [27]	✓	✗	✗	✗	✓	✓	✓	✗	✗
Transin [28]	✓	✓	✗	✗	✓	✗	✓	✓	✗
Wang [29]	✓	✗	✗	✗	✓	✓	✓	✗	✗
Winkel [30]	✗	✗	✗	✗	✓	✓	✓	✗	✗
Woźnicki [31]	✓	✗	✗	✗	✓	✓	✓	✗	✗
Zhong [32]	✓	✗	✗	✗	✓	✓	✓	✗	✗
Lesion Localization and Classification (LL&C)									
Cao [33]	✓	✗	✗	✗	✓	✓	✓	✓	✗
Gaur [34]	✓	✓	✓	✓	✓	✓	✓	✓	✗
Giannini [35]	✓	✗	✗	✗	✓	✓	✓	✓	✗
Greer [36]	✓	✗	✗	✓	✓	✓	✓	✓	✗
Litjens [37]	✓	✗	✗	✗	✓	✓	✓	✓	✗
Mehralivand [38]	✓	✓	✓	✓	?	?	✓	✓	✗
Schelb [39]	✓	✗	✗	✗	✓	✓	✓	✓	✓
Schelb [40]	✓	✗	✗	✗	✓	✓	✓	✓	✓
Thon [41]	✓	✓	✗	✗	?	✓	✓	✓	✓ *
Zhu [42]	✓	✗	✗	✗	✓	✓	✓	✓	✗
Patient Classification (PAT-C)									
Deniffel [43]	✓	✗	✗	✗	✓	✓	✓	✓	✗

(✓—Yes, ✗—No, ?—Unclear, AI—Artificial Intelligence). * commercially available.

**Table 4:** Per-Lesion Performance Comparison with 95% Confidence Intervals for Readers, CAD Systems and in Combination.

Study	Endpoint	Level	Zone	Readers(s) Alone				CAD System Alone				Combination			
				Cut-Off	SN %	SP %	AUC	Chosen Threshold	SN %	SP %	AUC	Interaction	SN %	SP %	AUC
ROI Classification (ROI-C)															
Algohary [19]	D’Amico ≥ Intermediate	Lesion	WP	PI-RADSv2, ≥3	71 (61–80)	67 (52–80)	NR	NR	63 (52–72)	91 (79–98)	0.75	NA	NA	NA	NA
Antonelli [20]	GS 3 + 3 vs. 4 component	Index Lesion	PZ	Suspected GS ≥ 3 + 4	72	40	NR	Matched to reader SP in training set	90	65	0.83	NA	NA	NA	NA
			TZ		82	44	NR		92	56	0.75	NA	NA	NA	NA
Bonekamp [21]	GS ≥ 3 + 4	Lesion	WP	PI-RADSv2, ≥4	88 (77–95)	50 (42–58)	NR	Matched to reader SN in training set	97 (88–100)	58 (50–66)	0.88	NA	NA	NA	NA
Dinh [23]	GS ≥ 3 + 4	Lesion	WP	Likert (1–5), ≥3	100 (100–100)	14 (8–19)	0.84 (0.77–0.89)	CAD SN of 95% in training set	96 (91–100)	**44 (36–52)**	0.88 (0.82–0.93)	NA	NA	NA	NA
Dikaios [22]	GS ≥ 3 + 4 or CCL ≥ 4 mm	Lesion	TZ	PI-RADSv1, ≥3	92 (74–99)	37 (25–50)	0.74 (0.63–0.86)	Probability threshold > 0.5	60	73	0.67 (0.55–0.79)	NA	NA	NA	NA
Hambrock [24]	GS ≥ 3 + 3 and >0.5 cm^3^	Lesion	WP	Likelihood scale (0–100), no cut-off	NR	NR	0.88 (0.85–0.93)	NA	NR	NR	0.90 (0.83–0.96)	CAD scores available to radiologist for interpretation	NR	NR	0.91 (0.86–0.97)
Iyama [25]	GS ≥ 3 + 4 and >10 mm vs. BPH	Lesion	TZ	PI-RADSv2, no cut-off	NR	NR	0.87 (0.81–0.93)	NA	NR	NR	**0.97 (0.94–0.99)**	NA	NA	NA	NA
Li [45]	GS ≥ 3 + 3	Index Lesion	WP	PI-RADSv2.1, ≥4	91	68	0.85	NR	82	82	0.86 (0.75–0.94)	LR model of PI-RADS, CAD score and PSA	79	96	**0.94**
Litjens [44]	GS ≥ 3 + 4	Lesion	WP	PI-RADSv1, ≥3	100 (98–100)	9 (0–19)	0.78 (0.70–0.85)	NR	NA	NA	NA	LR model of PI-RADS and CAD score	99 (98–100)	**26** **(0–60)**	**0.87** **(0.81–0.93)**
Niaf [26]	GS ≥ 3 + 3 and >2 × 2 mm in-plane	Lesion	PZ	Likelihood score (0–4), no cut-off	NR	NR	0.87 (0.81–0.92)	NA	NR	NR	0.82 (0.73–0.90)	CAD scores available to radiologist for interpretation	NR	NR	0.89 (0.83–0.94)
Niu [27]	GS ≥ 3 + 4	Lesion	PZ	PI-RADSv2, ≥4	79	75	0.76 (0.74–0.83)	NR	**87**	**89**	**0.89** **(0.82–0.94)**	NA	NA	NA	NA
			TZ		73	77	0.73 (0.69–0.81)	NR	**88**	**81**	**0.87** **(0.81–0.92)**	NA	NA	NA	NA
Transin [28]	GS ≥ 3 + 4	Lesion	PZ	PI-RADSv2, ≥3	97 (93–100)	37 (22–52)	0.74 (0.62–0.86)	CAD SN of 95% in training set	**89 (82–97)**	42 (26–58)	0.78 (0.69–0.87)	NA	NA	NA	NA
Wang [29]	GS ≥ 3 + 3 and >0.5 cm^3^	Index Lesion	WP	PI-RADSv2, ≥3	76 (67–84)	91 (87–94)	0.86 (0.83–0.90)	Youden statistics on test set	**90** **(84–95)**	88 (85–93)	**0.95** **(0.93–0.97)**	SVM model of PI-RADS and CAD score	**92** **(87–96)**	95 (93–99)	**0.98** **(0.95–0.99)**
Winkel [30]	GS ≥ 3 + 4	Lesion	PZ	PI-RADSv2, ≥3	100	53	0.60	NR	100	58	**0.90**	NA	NA	NA	NA
Woźnicki [31]	GS ≥ 3 + 4	Index Lesion	WP	PI-RADSv2, ≥4	NR	NR	0.69 (0.43–0.89)	NA	NA	NA	NA	Radiomics model ensembled with PI-RADS, PSAd and DRE models	NR	NR	0.84 (0.60–1.00)
Zhong [32]	GS ≥ 3 + 4	Lesion	WP	PI-RADSv2, ≥4	86	48	0.71 (0.58–0.85)	Point of best accuracy in test set	64	80	0.73 (0.58–0.88)	NA	NA	NA	NA
Lesion Localization and Classification (LL&C)															
Cao [33]	GS ≥ 3 + 4	Lesion	WP	PI-RADSv2, ≥3	81	NR	NR	FP per patient in test set matched to radiologist (0.62)	79	NR	0.81	NA	NA	NA	NA
Gaur [34]	GS ≥ 3 + 3	Index Lesion	WP	PI-RADSv2, ≥3	78	NR	NR	NR	NA	NA	NA	CAD identified lesions reviewed by radiologist	68	NR	NR
Giannini [35]	GS ≥ 3 + 4	Lesion	WP	PI-RADSv2, ≥3 and max diameter ≥7 mm	72 (61–81)	NR	NR	Voxel likelihood of malignancy ≥60% and lesion candidate ≥ 100 voxels in size	81 (61–93)	NR	NR	CAD identified lesions reviewed by radiologist	76 (65–85)	NR	NR
Greer [36]	GS ≥ 3 + 3	Index Lesion	WP	PI-RADSv2, ≥3	78 (69–85)	NR	NR	NR	NA	NA	NA	CAD identified lesions reviewed by radiologist	78 (69–86)	NR	NR
Mehralivand [38]	GS ≥ 3 + 4	Lesion	WP	PI-RADSv2, ≥3	51 (46–57)	NR	0.75	NR	NA	NA	NA	CAD identified lesions reviewed by radiologist	52 (45–61)	NR	0.78
Schelb [39]	GS ≥ 3 + 4	Sextant	WP	Mix of PI-RADSv1/v2, ≥3	67 (55–78)	68 (62–73)	NR	Point that most closely matched PI-RADS ≥ 3 performance in training set	59 (47–70)	66 (61–72)	NR	NA	NA	NA	NA
Schelb [40]	GS ≥ 3 + 4	Sextant	WP	PI-RADSv2, ≥3	71 (65–76)	62 (60–65)	NR	Iterative dynamic threshold that most closely matches PI-RADS ≥ 3 performance in most recent cases	70 (64–75)	66 (63–69)	NR	NA	NA	NA	NA
Thon [41]	GS ≥ 2 + 3	Lesion	WP	PI-RADSv2, no cut-off	NR	NR	0.68 (0.59–0.76)	Youden statistics on test set	47	75	0.64 (0.53–0.75)	NA	NA	NA	NA
Zhu [42]	GS ≥ 3 + 4	Lesion	WP	PI-RADSv2, ≥3	77 (68–84)	NR	NR	NR	NA	NA	NA	Radiologist reported with but not limited by CAD probability map	**89** **(82–94)**	NR	NR

(AUC—Area under the receiver operating characteristic curve; BPH—benign prostatic hyperplasia; CAD—computer-aided diagnosis; CCL—cancer core length; FP—false positive; GS—Gleason score; LR—logistic regression; NR—not reported; PI-RADS—Prostate Imaging-Reporting and Data System; PZ—peripheral zone; ROI- region of interest; SN—sensitivity; SP—specificity; SVM—support vector machine; TZ—transition zone; WP—whole prostate). Bold results indicate statistically significant differences to that of reader(s) alone, *p*-value < 0.05.

**Table 5:** Per-Patient Performance Comparison with 95% Confidence Intervals for Readers, CAD Systems and in Combination.

Study	Endpoint	Zone	Reader(s) Alone				CAD System Alone				Combination			
			Cut-Off	SN %	SP %	AUC	Chosen Threshold	SN %	SP %	AUC	Interaction	SN %	SP %	AUC
ROI Classification (ROI-C)														
Bonekamp [21]	GS ≥ 3 + 4	WP	PI-RADSv2, ≥4	89 (76–96)	43 (33–54)	NR	Matched to reader SN in training set	96 (85–99)	51 (40–62)	NR	NA	NA	NA	NA
Dinh [23]	GS ≥ 3 + 4	WP	Likert (1–5), ≥3	100 (100–100)	9 (2–15)	0.88 (0.68–0.96)	CAD SN of 95% in training set	100 (100–100)	**40** **(28–51)**	**0.95** **(0.90–0.98)**	NA	NA	NA	NA
Woźnicki [31]	GS ≥ 3 + 4	WP	PI-RADSv2, ≥4	91 (82–98)	28 (13–46)	NR	NR	NA	NA	NA	Radiomics model ensembled with PI-RADS, PSAd and DRE models	91 (81–98)	57 (38–74)	NR
Lesion Localization and Classification (LL&C)														
Gaur [34]	GS ≥ 3 + 3	WP	PI-RADSv2, ≥3	94 (91–96)	45 (38–52)	0.82	NR	NA	NA	NA	CAD identified lesions reviewed by radiologist	**82** **(75–88)**	**72** **(63–80)**	0.83
Giannini [35]	GS ≥ 3 + 4	WP	PI-RADSv2, ≥3 and max diameter ≥7 mm	81 (70–90)	75 (68–92)	NR	NR	**96** **(78–100)**	NR	NR	CAD identified lesions reviewed by radiologist	**91** **(82–97)**	78 (71–85)	NR
Greer [36]	GS ≥ 3 + 3	WP	PI-RADSv2, ≥3	91 (87–95)	70 (62–79)	0.88 (0.83–0.92)	NR	NA	NA	NA	CAD identified lesions reviewed by radiologist	90 (85–95)	**57** **(47–66)**	0.85 (0.79–0.90)
Litjens [37]	GS ≥ 3 + 4 *	WP	PI-RADSv1, ≥3	≈100 ^†^	≈52 ^†^	NR	NA	NR	NR	0.83	NA	NA	NA	NA
Mehralivand [38]	GS ≥ 3 + 4	WP	PI-RADSv2, ≥3	82	NR	0.82	NR	NA	NA	NA	CAD identified lesions reviewed by radiologist	84	NR	0.78
Schelb [39]	GS ≥ 3 + 4	WP	Mix of PI-RADSv1/v2, ≥3	96 (80–100)	22 (10–39)	NR	Point that most closely matched PI-RADS ≥3 performance in training set	96 (80–100)	31 (16–48)	NR	NA	NA	NA	NA
Schelb [40]	GS ≥ 3 + 4	WP	PI-RADSv2, ≥3	98 (94–100)	17 (11–24)	NR	Iterative dynamic threshold that most closely matches PI-RADS ≥ 3 performance in most recent cases	99 (95–100)	24 (17–31)	NR	NA	NA	NA	NA
Zhu [42]	GS ≥ 3 + 4	WP	PI-RADSv2, ≥3	84 (75–91)	56 (43–69)	0.83 (0.76–0.88)	NR	NA	NA	NA	Radiologist reported with but not limited by CAD probability map	**93** **(86–98)**	66 (53–77)	**0.89** **(0.83–0.94)**
Patient Classification (PAT-C)														
Deniffel [43]	GS ≥ 3 + 4	WP	PI-RADSv2, ≥3 and PSAd ≥0.15 ng/mL^2^	95 (84–100)	35 (19–52)	NR	CSPCa likelihood ≥ 0.2	100 (100–100)	52 (32–68)	0.85 (0.76–0.97)	NA	NA	NA	NA

(AUC—area under the receiver operating characteristic curve; CAD—computer-aided diagnosis; GS—Gleason score; NR—not reported; PI-RADS—Prostate Imaging-Reporting and Data System; ROI—region of interest; SN—sensitivity; SP—specificity; WP—whole prostate). Bold results indicate statistically significant differences to that of the reader(s) alone, *p*-value >0.05. * 3 + 4 vs. benign, Gleason 3 + 3 excluded. † Approximate values derived from study figures.

## Supplementary Materials

The following are available online at https://www.mdpi.com/article/10.3390/cancers13133318/s1, Supplementary Material: Adapted QUADAS-2 Tool with Additional Signaling Questions.

## Appendix A

**Table A1:** MEDLINE Search Terms and Strategy.

#	Search Term
1	exp Prostatic Neoplasms/
2	Prostat * Cancer *.mp.
3	Prostat * Neoplasm *.mp.
4	Prostat * Malignanc *.mp.
5	Prostat * Tumo ?r.mp.
6	Prostat * carcinoma *.mp.
7	1 or 2 or 3 or 4 or 5 or 6
8	exp Magnetic Resonance Imaging/
9	Magnetic Resonance Imaging.mp.
10	Magnetic Resonance.mp.
11	MRI.mp.
12	MR.mp.
13	8 or 9 or 10 or 11 or 12
14	exp Artificial Intelligence/
15	exp Diagnosis, Computer-assisted/
16	Artificial Intelligence.mp.
17	AI.mp.
18	Computer Assisted.mp.
19	Computer Diagnosis.mp.
20	Computer Aided Diagnosis.mp.
21	Computer Aided Detection.mp.
22	CAD *.mp.
23	Machine Learning.mp.
24	Deep Learning.mp.
25	Neural Network *.mp.
26	Convolutional Neural Network *.mp.
27	CNN.mp.
28	Support Vector Machine *.mp.
29	SVM.mp.
30	Automatic Classif *.mp.
31	14 o 15 or 16 or 17 or 18 or 19 or 20 or 21 or 22 or 23 or 24 or 25 or 26 or 27 or 28 or 29 or 30
32	7 and 13 and 31
33	32 Limit to Human Studies
34	33 Limit to English Language

*—MEDLINE truncation to find variant word endings, ?—MEDLINE wildcard to find alternate spellings.
