# Supplementary material for: Artificial Intelligence Compared to Radiologists for the Initial Diagnosis of Prostate Cancer on Magnetic Resonance Imaging: A Systematic Review and Recommendations for Future Studies
Source: Cancers (Basel). 2021 Jul 1;13(13):3318. doi: 10.3390/cancers13133318 (PMC8268820; doi:10.3390/cancers13133318)
Supplement: Supplementary file 1 [file cancers-13-03318-s001.zip › cancers-1258492-supplementary.pdf]

cancers

MDPI

Systematic Review

Artificial Intelligence Compared to Radiologists for the Initial Diagnosis of Prostate Cancer on Magnetic Resonance Imaging: A Systematic Review and Recommendations for Future Studies

Tom Syer [1,†], Pritesh Mehta [2,†], Michela Antonelli [3], Sue Mallett [1], David Atkinson [1], Sébastien Ourselin [3] and Shonit Punwani [1,*]

1 Centre for Medical Imaging, Division of Medicine, Bloomsbury Campus, University College London, London WC1E 6DH, UK; t.syer@ucl.ac.uk (T.S.); sue.mallett@ucl.ac.uk (S.M.); d.atkinson@ucl.ac.uk (D.A.)
2 Department of Medical Physics and Biomedical Engineering, Faculty of Engineering Sciences, Bloomsbury Campus, University College London, London WC1E 6DH, UK; pritesh.mehta.17@ucl.ac.uk
3 School of Biomedical Engineering & Imaging Sciences, Faculty of Life Sciences and Medicine, St Thomas' Campus, King's College London, London SE1 7EH, UK; michela.antonelli@kcl.ac.uk (M.A.); sebastien.ourselin@kcl.ac.uk (S.O.)
* Correspondence: shonit.punwani@gmail.com
† Joint First Author.

Citation: Syer, T.; Mehta, P.; Antonelli, M.; Mallett, S.; Atkinson, D.; Ourselin, S.; Punwani, S. Artificial Intelligence Compared to Radiologists for the Initial Diagnosis of Prostate Cancer on Magnetic Resonance Imaging: A Systematic Review and Recommendations for Future Studies. *Cancers* **2021**, *13*, 3318. https://doi.org/10.3390/cancers13133318

Academic Editors: Reza Forghani, Rajiv Gupta and Farhad Maleki

Received: 28 May 2021
Accepted: 30 June 2021
Published: 1 July 2021

Publisher's Note: MDPI stays neutral with regard to jurisdictional claims in published maps and institutional affiliations.

Copyright: © 2021 by the authors. Submitted for possible open access publication under the terms and conditions of the Creative Commons Attribution (CC BY) license (http://creativecommons.org/licenses/by/4.0/).

Supplementary material. Adapted QUADAS-2 Tool with Additional Signaling Questions.

Patient Selection

Was a consecutive or random sample of patients enrolled?
Was a case-control design avoided?
Did the study avoid inappropriate exclusions?
Was the intention for patients either to receive all index tests or to be randomly allocated to index tests?
If patients were randomized was the allocation sequence random?
If patients were randomized was the allocation sequence concealed until patients were enrolled and assigned to index tests?
Could the selection of patients have introduced bias in the comparison?
Are there concerns that the included patients do not match the review question?

Index tests

Were the index test(s) interpreted without knowledge of the results of the reference standard?
If a threshold was used, was it pre-specified?
If patients received multiple index tests, were the test results interpreted without knowledge of the results of the other index test(s)?
If patients received multiple index tests, is undergoing one index test unlikely to affect the performance of the other index tests(s)?
Could the conduct or interpretation of the index tests have introduced bias in the comparison?
Are there concerns that the index test(s), its conduct, or interpretation differ from the review question?

Reference standard

Is the reference standard/ground truth likely to correctly classify the target condition?
Did the reference standard avoid incorporating any of the index tests?

Could the reference standard, its conduct or its interpretation have introduced bias in the comparison?

Are there concerns that the target condition as defined by the standard does not match the review question?

Flow and timing

Was there an appropriate interval between index tests and reference standard?
Did all patients receive a reference standard?
Did all patients receive the same reference standard?
Was the same reference standard used for all index tests?
Were all patients included in the analysis?
Are the proportion and reasons for missing data similar across index tests?
Could the patient flow have introduced bias in the comparison?

AI in radiology specific quality assessment questions

1. Are all three image sets (training, validation, and test sets) defined?
2. Is an *external* test set used for final statistical reporting?
3. Have multivendor images been used to evaluate the AI algorithm?
4. Are the sizes of the training, validation and test sets justified?
5. Was the AI algorithm trained using a standard of reference that is widely accepted in our field?
6. Was preparation of images for the AI algorithm adequately described?
7. Were the results of the AI algorithm compared with expert radiologists and/or pathology?
8. Was the manner in which the AI algorithm makes decisions demonstrated?
9. Is the AI algorithm publicly available?
